# The Expression Pattern of Insulin-Like Growth Factor Subtype 3 (igf3) in the Orange-Spotted Grouper *Epinephelus coioides* and Its Function on Ovary Maturation

**DOI:** 10.3390/ijms24032868

**Published:** 2023-02-02

**Authors:** Fang Jiao, Bing Fu, Yan Yang, Huayi Xue, Yuanyuan Wu, Huihong Zhao, Qing Wang, Huirong Yang

**Affiliations:** 1 College of Marine Sciences, South China Agricultural University, Guangzhou 510642, China; 2 Zhongshan Innovation Center, South China Agricultural University, Zhongshan 528400, China

**Keywords:** *igf3*, Epinephelus coioides, ovarian, sex differentiation

## Abstract

A new insulin-like growth factor (Igf) subtype 3 (*igf3*) has recently been found in the bony fish orange-spotted grouper (*Epinephelus coioides*). However, the role of *igf3* in the maturation of the ovary and sex differentiation in *E. coioides* is currently unknown. We examined the ovarian localization and receptor binding of the novel ortholog Igf3 using qRT-PCR, and Western blotting, combined with in situ hybridization and immunohistochemistry methods. Results demonstrated the presence of *igf3* mRNA and protein in mature oocytes. Furthermore, Igf3 protein expression was not detected in testis, brain, kidney and liver homogenates. The calculated molecular weight of Igf3 was 22 kDa, which was consistent with the deduced amino acid sequence from the full-length open reading frame. The immunoreactivity showed that Igf3 was strongly present in the follicle staining fully-grown stage. The *igf3* mRNA expression level was significantly positively correlated with ovarian follicular maturation. Meanwhile, Igf3 increased germinal-vesicle breakdown in a time- and dose-dependent manner. In vitro, treatment of primary ovarian cells with Igf3 up-regulated significantly the mRNA expression level of genes related to sex determination and reproduction such as forkhead boxl2 (*foxl2*), dosage-sensitive sex reversal adrenal hypoplasia critical region on chromosome x gene 1 (*dax1*), cytochrome P450 family 19 subfamily member 1 a (*cyp19a1a*), cytochrome P450 family 11 subfamily a member 1 a (*cyp11a1a*) and luteinizing hormone receptor 1 (*lhr1*). Overall, our results demonstrated that *igf3* promotes the maturation of the ovary and plays an important role in sex differentiation in *E. coioides*.

## 1. Introduction

The insulin-like growth factor (Igf) system is an evolutionary-conserved complex that regulates cell proliferation in a variety of contexts, including growth, proliferation, survival, migration, and differentiation [1,2,3,4,5,6]. Previous studies on the functions of the Igf system in fish mainly focused on Igf1 and Igf2 [7,8,9,10,11,12,13]. Igf1 and Igf2 have been identified in fish ovaries. Indeed, *igf1* expression has been reported in granulosa cells, yolk, and mature ovarian membrane cells during the initial stage of gonadal development. The *igf2* mRNA and protein are localized to the granulosa cell layer of the late follicular [14,15,16]. However, *igf1* and *igf2* mRNA are also found in other organs, from unfertilized eggs to adult fish. The functional analysis of Igf1 and Igf2 revealed that the two genes promote growth and maturation in killifish (*Fundulus grandis*), short-finned eel (*Anguilla australis*), zebrafish (*Danio rerio*) and striped bass (*Lateolabrax japonicas*) [17,18,19,20]. Therefore, Igf1 and Igf2 are important enzymes in the reproduction and growth of fish.

Besides Igf1 and Igf2, fish also contain *igf3* as an important enzyme in reproduction and growth. Furthermore, *igf3* has been found in tilapia (*Oreochromis niloticus*), *D. rerio*, *E. coioides* and common carp (*Cyprinus carpio*) [1,8,21,22]. In *D. rerio*, the *igf3* mRNA level was relatively low in early follicular but higher in mature follicular [21]. Functional analyses revealed that *igf3* promotes the development, reproduction and migration of germ cells [21,23,24]. Accordingly, incubating oocytes using Igf3 promoted germinal vesicle breakdown (GVBD) and ovarian blastocyst rupture [21]. Moreover, in an in vitro study, the synthesis and expression of Igf3 were up-regulated when human chorionic gonadotropin (hCG) was added to the follicle [17,21]. Furthermore, *Igf3* mRNA level increased in *D. rerio* both in oocytes and follicular, suggesting its potential role in the regulation of gonadal differentiation and ovulation cycle [21]. These studies demonstrate that Igf3 plays an important role in fish reproduction. 

Igf3 regulates several genes involved in ovary maturation. Accordingly, treating ovarian and testicular primary cells with Igf3 up-regulated the expression of forkhead boxl2 (*foxl2*), dosage-sensitive sex reversal adrenal hypoplasia critical region on chromosome x gene 1 (*dax1*), splicing-transcription factor 1 (*sf1*), cytochrome P450 family 19 subfamily member 1 a (*cyp19a1a*), cytochrome P450 family 11 subfamily a member 1 a (*cyp11a1*) as well as cytochrome P450 family 11 subfamily b member 2 (*cyp11b2*) in a time- and dose-dependent manner in *O. niloticus* [2]. However, studies describing the precise role of Igf3 in the regulation of gonadal differentiation are currently limited [21]. Consequently, little direct evidence exists on the role of Igf3 in regulating ovary maturation. The scarcity of information limits our understanding of the role of Igf3 in various fish species. 

Therefore, we examined the expression pattern of *igf*3 mRNA and protein orange-spotted grouper *E. coioides* by using five stages of follicular. The effect of Igf3 on ovarian maturation was studied by in vitro incubation of Igf3 protein and its regulatory role on genes related to sex determination and reproduction such as *foxl2*, *dax1*, *cyp19a1a*, *cyp11a1* and *cyp11b2*. Furthermore, we examined the role of *igf3* on reproductive development and sex determination in *E. coioides*. The obtained results provide a reference for understanding the role of *igf3* in maturation regulation in fish.

## 2. Results

### 2.1. Distribution of igf3 mRNA in the Developing Ovary

**Figure 1:**
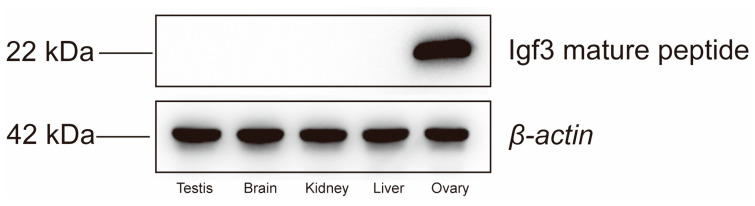
Western blot verification of Igf3.

**Figure 2:**
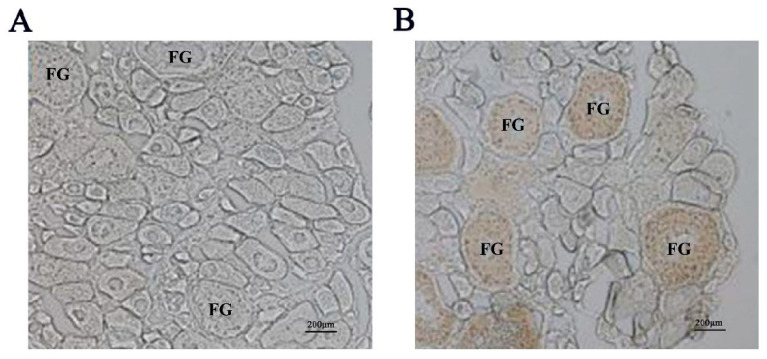
Immunohistochemical detection of Igf3 in *E. coioides* ovarian tissues. (**A**) negative control lacking primary antibody. (**B**) Igf3-specific polyclonal primary antibody. Note: FG indicates full grown stages of follicular.

**Figure 3:**
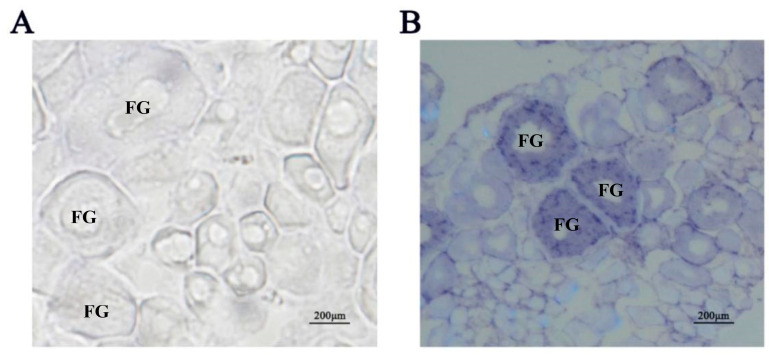
Distribution of *igf3* mRNA in *E. coioides* ovaries from frozen tissue sections. (**A**) sense probe. (**B**) antisense probe. Note: FG indicates full grown stages of follicular.

We initially detected expression of the Igf3 protein in ovary tissue homogenates by Western blot analysis. In contrast, we could not detect the presence of Igf3 in testis, brain, kidney and liver homogenates (Figure 1 and Figure S1). The calculated molecular weight of Igf3 was 22 kDa, which was consistent with the deduced amino acid sequence from the full-length ORF of Igf3. This antibody was further used for the immunohistochemistry experiment. We observed signals only in the presence of the Igf3-specific antibody. The strong immunoreactivity showed that the follicle staining in the FG stage was the strongest (Figure 2). We also examined *igf3* mRNA expression in frozen sections of *E. coioides* ovaries. Tissues treated with sense RNA probes lacked staining, indicating that our probes were specific for *igf3* mRNA (Figure 3A). We detected positive signals in follicular FG and MV stages, with FG stage follicular staining strong, but early stages had weak signals (Figure 3B). 

### 2.2. Expression of igf3 mRNA in Different Stages of Ovarian Follicular

**Figure 4:**
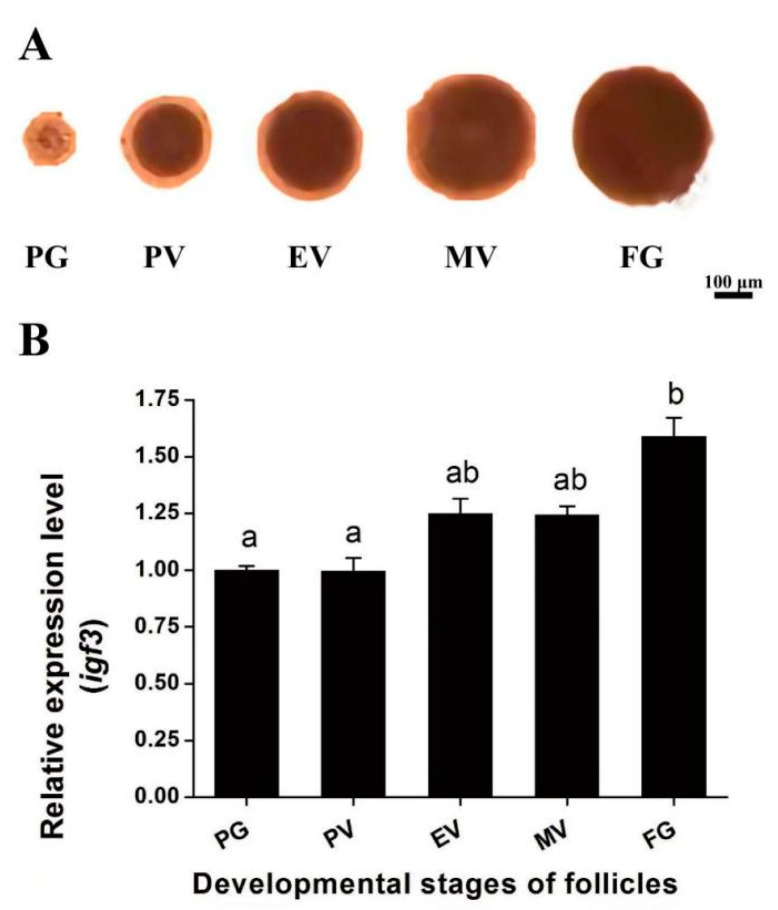
Stage-dependent expression of *igf3* in developing *E. coioides* follicles. (**A**) Morphology of *E. coioides* follicular at different stages of maturation. PG—primary growth; PV—previtellogenic; EV—early vitellogenic; MV—midvitellogenic; FG—full-grown. (**B**) Expression of *igf3* mRNA during different stages of ovarian follicle development. Different letters (a and b) indicate significant differences.

Ovarian follicular were examined at different stages of development, and we followed the accumulation of *igf3* mRNA as development proceeded through the PG, PV, EV, MV and FG stages. The *igf3* steady-state mRNA level correlated well with this progression (Figure 4). 

### 2.3. Igf3 Promotes the Development and Maturation of Follicular

**Figure 5:**
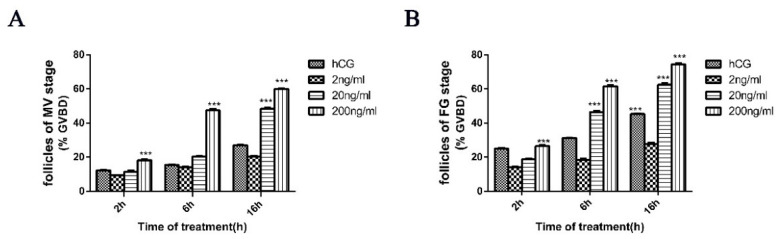
Germinal vesicle breakdown (GVBD) alterations in midvitellogenic (MV) and fully-grown (FG) stages of follicular. Igf3 was added at the (**A**) MV and (**B**) FG stages. (*n* = 4). ***, *p* < 0.001.

**Figure 6:**
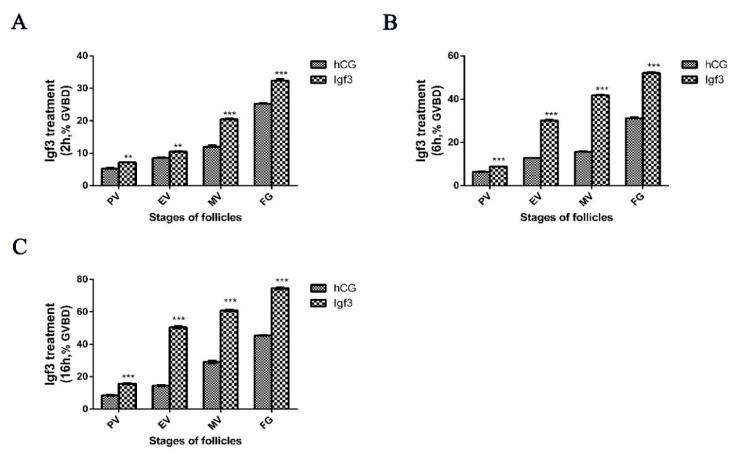
Effects of Igf3 on follicular Germinal vesicle breakdown (GVBD) in the previtellogenic (PV), early vitellogenic (EV), midvitellogenic (MV) and fully-grown (FG) stages of development. Cells were treated for (**A**) 2, (**B**) 6 and (**C**) 16 h. (*n* = 4). **, *p* < 0.01, ***, *p* < 0.001.

We utilized recombinant Igf3 to determine whether this could alter ovarian follicle development when added externally to follicular at the MV and FG phases of development. At a low (2 ng/mL) Igf3 level, the GVBD of follicular in neither MV nor FG significantly changed over time. At higher (20 ng/mL) concentration, the GVBD of MV phase cells was significantly up-regulated after 16 h, and for the FG phase, this occurred after 6 and 16 h. Under high level (200 ng/mL) Igf3, GVBD of follicular in MV and FG phases reached levels of significance at all three treatment times (Figure 5). Therefore, we extended the experiment and treated and examined the level of GVBD of follicular in the PV, EV, MV and FG phases by using 200 ng/mL Igf3. In all the developmental phases, Igf3 induced GVBD. However, the effects were more pronounced in the more mature follicular and as the exposure time increased (Figure 6).

### 2.4. Effects of Igf3 on the Expression of Genes Related to Sex Determination and Reproduction

**Figure 7:**
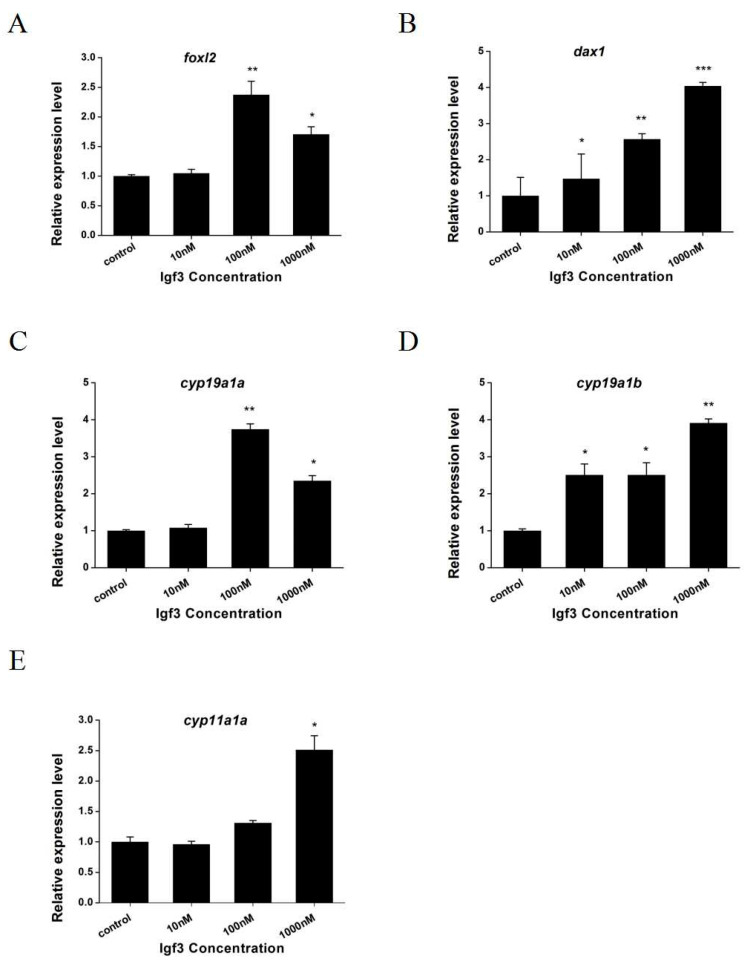
Igf3 added to primary ovarian cells increases expression of sex differentiation-related genes. (**A**) forkhead boxl2—*foxl2*; (**B**) dosage-sensitive sex reversal adrenal hypoplasia critical region on chromosome x gene 1—*dax1*; (**C**) cytochrome P450 family 19 subfamily member 1 a—*cyp19a1a*; (**D**) cytochrome P450 family 19 subfamily a1 member b—*cyp19a1b*; and (**E**) cytochrome P450 family 11 subfamily b member 2—*cyp11a1.* (*n* = 4). *, *p* < 0.05, **, *p* < 0.01, ***, *p* < 0.001.

**Figure 8:**
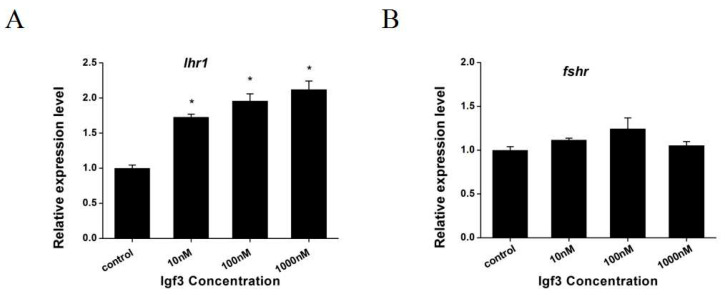
Effects of Igf3 treatment on primary ovarian cells on the expression of reproduction-related genes. (**A**) *ihr1* and (**B**) *fshr* at the indicated dosages. (*n* = 4). *, *p* < 0.05.

We examined whether Igf3 added to primary ovary cells altered the expression of *sf1* and related genes. We measured steady-state mRNA levels in the primary ovary and found that *foxl2*, *dax1*, *cyp19a1a*, *cyp19a1b* and *cyp11a1* were up-regulated when Igf3 was added to the cell culture. However, expression levels were dose-dependent such that *foxl2* and *cyp19a1a* genes increased significantly only with 100 and 1000 nM Igf3 (Figure 7A,C). The expression of *dax1* and *cyp19a1b* genes increased significantly under all levels of Igf3 (Figure 7B,D). The expression of the *cyp11a1* gene increased significantly only with Igf3 at 1000 nM (Figure 7E). In contrast, Igf3 addition increased the mRNA expression level for luteinizing hormone receptor 1 (*lhr1*) in a dose-independent manner. However, the mRNA levels for follicle-stimulating hormone receptor (*fshr*) remained unchanged at basal levels with Igf3 addition (Figure 8). 

## 3. Discussion

A novel Igf3 has been described in the ovaries of *O. niloticus* and *D. rerio* [1,2,14]. In this study, the Igf3 protein was expressed only in the ovary. We confirmed that Igf3 was not present in the testis, brain, kidney and liver tissues in *E. coioides*, similar to previous results in *D. rerio* [21]. This indicated that the polyclonal antibody could recognize the endogenous Igf3 protein with high specificity. Furthermore, these results confirmed that the Igf3 mature peptide was ovary-specific in *E. coioides*.

We found both *igf3* mRNA and Igf3 in FG and MV stage follicular by in situ hybridization and immunohistochemical. Previous studies detected *igf3* mRNA in the follicular cell layer of *O. niloticus* and *D. rerio* ovaries [1,21]. In these studies, *igf3* mRNA was relatively low in the early follicular but increased significantly after the MV stage follicular and was high in the FG follicular [1,21]. In addition, the Igf3 protein from *C. carpio* was also located in ovarian granulosa cells [8]. These suggest that *igf3* plays a regulatory role in *E. coioides* ovary maturation. Together, these results indicated that Igf3 is an ovarian-specific growth factor that is active in the late stages of follicular development. Furthermore, these studies show that *igf3* mainly exists in more mature ovarian follicular and has a certain relationship with the maturation of the ovary.

To explore the functional roles of *igf3* in the ovary maturation of *E. coioides*, we separated follicular at different stages and analyzed its temporal expression pattern in follicular of different stages by using qRT-PCR. We found that *igf3* mRNA level during *E. coioides* follicular development increased from the early stages and was maximal at FG, similar to the results in *D. rerio* [21,25]. Interestingly, the *igf3* expression pattern was similar to that of *lhr* [21,26]. This demonstrated that *igf3* mRNA expression was linked to and dependent on the developmental stage. This implies that *igf3* may be involved in ovarian maturation. The addition of Igf3 also induced GVBD in ovarian follicular of *E. coioides* in a time- and dose-dependent manner. This further indicates that Igf3 is involved in the regulation of *E. coioides* ovarian maturation. Igf1 induced GVBD in ovarian follicular of red seabream (*Pagrus major*), sea perch (*Lateolabrax japonicus*), white bass (*Morone saxatilis*), *C. carpio, D. rerio* and southern flounder (*Paralichthys lethostigma*) [12,20,27,28,29,30]. Moreover, Igf2 induced GVBD in the ovarian follicular of *C. carpio*, *D. rerio* and *P. lethostigma* [20,29,30]. We found similar effects for Igf3. These results show that Igf3 plays an important role in the maturation of *E. coioides* oocytes.

*Foxl2* and *dax1* are factors related to female sex differentiation, which cause animal gonads to differentiate towards ovaries [31,32,33]. *Cyp19a1a* and *cyp19a1b* are aromatase genes that induce estrogen production [34,35]. *Cyp11a1* is a kind of steroidase gene that catalyzes the conversion of cholesterol to pregnenolone [36]. In addition, estrogen is involved in the sex differentiation mechanism of bony fish [32,37]. In this study, we found that Igf3 protein addition to primary ovary cells increased *foxl2*, *dax1*, *cyp19a1a*, *cyp19a1b* and *cyp11a1* mRNA expression levels. This result indicates that Igf3 regulates the expression of sex-related genes and steroidase-related genes in the ovaries of *E. coioides*. Therefore, it is speculated that Igf3 participates in the sex determination of *E. coioides* and regulates its sex differentiation. In *O. niloticus*, Igf3 up-regulated the expression of *foxl2*, doublesex and mab-3-related transcription factor 1(*dmrt1*), *cyp11a1* and *cyp19a1a* in a dose-dependent manner [2]. It further proves that Igf3 plays an important role in fish sex differentiation. In *D. rerio,* Igf3 was found to be a downstream factor of estrogen signaling in sex differentiation [38]. In addition, studies have shown that the expression level of *igf3* positively correlated with that of *cyp19a1a* and *foxl2*, which have been shown to play a key role in the regulation of *cyp19a1a* expression [32,38,39,40]. The somatic cell signaling pathway mediated by *foxl2* is a female differentiation pathway, and *foxl2* can regulate the expression of *cyp19a1*. Therefore, it is speculated that Igf3 acts on the upstream position of *foxl2* through a somatic signaling pathway mediated by *foxl2*. In the study, the expression of *foxl2* and *cyp19a1a* showed a dose-dependent increase and then declined. Therefore, it is preliminary guessed that there may be feedback regulation between Igf3 and the two. Taken together, we demonstrated that Igf3 is essential for ovary differentiation in *E. coioides*.

Both luteinizing hormone (LH) and follicle-stimulating hormone (FSH) are involved in reproductive processes and play key roles in germ cell growth and development [41]. Ovary cells treated with Igf3 enhanced their growth and differentiation, increased related hormone levels and accelerated ovarian maturation [24,42]. In this study, Igf3 promoted *lhr1* expression in a concentration-dependent manner but had no effect on *fshr* expression. This indicated that Igf3 regulated a gene related to reproduction in the ovary. Therefore, Igf3 was most likely involved in regulating the reproductive processes of *E. coioides* and promoted ovarian development and maturity. In a previous study, oogonia development into oocytes was inhibited by the Kit ligand A (*kitlga*), and when the *igf* system was inhibited in somatic cells, increased LH production hindered *kitlga* expression in *D. rerio* [43]. Our results indicated that Igf3 stimulates an increase in *lhr1* expression resulting in increased LH bound to its cognate receptor. This possibly led to the down-regulation of *kitlga* and accelerated oocyte maturation.

## 4. Materials and Methods

### 4.1. Fish, Cells Line and Igf3 Mature Peptide

The *E. coioides* (the female fish with a body length of 56.5 ± 5.5 cm and a body weight of 3.6 ± 0.6 kg) used in this study were collected from the Guangdong Marine Fisheries Test Center (N22°705093′, E114°541433′) Guangdong China. The fish were kept in a recirculating seawater system that was maintained at 25 °C for two weeks before the start of experiments. Tissue samples were collected from fish and stored at −80 °C immediately. Voucher specimens were deposited at the Department of Aquatic Animal Medicine, College of Marine Sciences, South China Agricultural University, Guangzhou, China (Accession number 201805021). Igf3 mature peptide was expressed in *Escherichia coli* and purified in the laboratory of College of Marine Sciences, South China Agricultural University, as described previously [44].

### 4.2. RNA Extraction, cDNA Synthesis and Real-Time Polymerase Chain Reaction (qRT-PCR)

Total RNA from liver, kidney, brain, testis and ovarian tissues was extracted using Trizol reagent (Invitrogen, Waltham, MA, USA). The total RNA from cells was extracted using a Takara MiniBEST Universal RNA Extraction Kit (Takara, Shiga, Otsu, Japan). Reverse transcription reactions were performed using an RNase-free DNase I kit (Fermentas, Waltham, MA, USA) using the protocol provided by the manufacturer. The *β-actin* was used as a reference gene for qRT-PCR gene targets. The primers used in this study are given in Table 1. Each sample was run in triplicate, and reactions without templates were used as negative controls. The qRT-PCR reaction was run on a Roche Light Cycler^®^ 480 System, and the relative quantification calculated using the ΔΔC_T_ method [45].

### 4.3. Ovarian Follicle Experiment

Mature female fish were anesthetized with 1:10,000 eugenol (purity 99%, Shanghai Reagent, China) to avoid stress before harvesting. The abdomens were incised, and a small part of ovarian tissue was sampled and placed into L15 medium (Thermo Scientific, Waltham, MA, USA) and dispersed in the culture dish. The ovarian follicular development stages were classified as described previously [21]. There are five stages of follicular development that can be readily assessed by observing follicular diameters as described previously [46,47]. The primary growth (PG), previtellogenic (PV), early vitellogenic (EV), midvitellogenic (MV) and fully-grown (FG) stages are represented by follicular with diameters of <100, 200, 250, 400 and 500 μm, respectively. PG, PV, EV, MV and FG phase cells were identified and divided into parallel stages. Igf3 was added to the follicular development stages at 2, 20 and 200 ng/mL and 20 IU/mL hCG was used as a control. Germinal vesicle breakdown (GVBD) was studied by using light microscopy after 2, 6 and 16 h of incubation. 

### 4.4. In Situ Hybridization

Intact ovaries from adult female *E. coioides* were dissected and fixed in 4% buffered paraformaldehyde overnight at 4 °C. The ovaries were then rinsed twice with cold phosphate-buffered saline (PBS), transferred into 30% sucrose solution for 48 h and stored at 4 °C for drying. The dried samples were frozen using dry ice and embedded in optimal cutting temperature (OCT) compound (Sakura, Torrance, CA, USA). The tissue was sliced at −21 °C, and sections were attached to cationic anti-off slides (Thermo Scientific, Waltham, MA, USA) and then covered and incubated at 42 °C for 1 to 2 d. A cDNA fragment of *igf3* was amplified by PCR (Table 1) and cloned into the pGEM-T easy transcription vector (Promega, USA). Sense and antisense *igf3* riboprobes were synthesized from the pGEM-T easy transcription vector construct containing *igf3* after linearization with either *Spe*I or *Aat*II. The RNA probes were labeled using the DIG RNA Labeling Kit (Roche, Germany). Tissue sections were first prehybridized for 30 min, and 250 μL of hybridization buffer containing 150 ng of DIG-labeled sense or antisense *igf3* riboprobe was added to each slide and incubated in a humidified box at 42 °C for 16 h. After hybridization, sections were sequentially washed twice in 2 × saline-sodium citrate (SSC) (1 × SSC = 0.15 M NaCl, 15 mM Na citrate) at room temperature for 15 min and then in 1 × SSC and 0.1 × SSC at 55 °C for 1 h. The hybridization signals were detected using anti-DIG conjugated with alkaline phosphatase and visualized with the nitroblue tetrazolium chloride/5-bromo-4chloro-3-indolyl phosphate substrate solution (Dongsheng, China). The sections were dehydrated through a graded ethanol and xylene series and photographed. In this process, T7 and SP6 promoters were introduced into the upstream primers of gene-specific oligonucleotides to enable direct in vitro transcription of purified PCR fragments [48]. Among them, the sections should be treated with alcohol before observation.

### 4.5. Western Blotting

Western blot analysis was performed using homogenized extracts of liver, kidney, brain, testis and ovarian tissue samples using RIPA buffer (Thermo Scientific, Waltham, MA, USA). Tissue proteins were separated by using 12% sodium dodecyl sulfate–polyacrylamide gel electrophoresis (SDS-PAGE) and electrotransferred to a polyvinylidene fluoride (PVDF) membrane (Thermo Scientific, Waltham, MA, USA). Primary anti-Igf and β-actin (Invitrogen, USA) antibodies were incubated with the blots at 1:1000 dilutions for 45 min. Secondary antibodies were incubated with homogenates from testis, brain, kidney, liver and ovarian tissues before adding to the primary antibody blots. Electrochemiluminescence (ECL) detection was performed to observe the results (Applygen, Haidian, Beijing, China).

### 4.6. Immunohistochemistry

Ovarian tissue samples from *E. coioides* were embedded in paraffin and sectioned. They were then subjected to an antigen retrieval step before proceeding as described previously [49]. The sections were then blocked with 5% bovine serum albumin (BSA) for 30 min and incubated with the primary antibody at 4 °C overnight. The sections were then allowed to stand at room temperature for 30 min, washed with PBS and the secondary antibody was added dropwise and incubated at 37 °C for 1 h following the recommendations of the manufacturer (Santa Cruz Biotechnology, Dallas, TX, USA). The sections were washed with PBS and developed using 3, 3′-diaminobenzidine and counterstained with hematoxylin. The sections were then dehydrated by using graded ethanol and xylene series and observed using light microscopy.

### 4.7. Statistical Analysis

Data were analyzed using one-way analysis of variance (ANOVA). Before that, all data were tested for normality using the Kolmogorov–Smirnov test and homogeneity of variances using Levene’s test. Results with *p*-values less than 0.05 were considered statistically significant. The results are presented as mean ± S.E.M. for four replicates for each fish. All analyses were performed using Statistical Package for Social Science (SPSS) for Windows (SPSS version 20, IBM, Armonk, NY, USA). 

## 5. Conclusions

In the study, *igf3* mRNA and Igf3 protein were abundantly expressed in the more mature follicular of *E. coioides*. Western blot analysis demonstrated that Igf3 was an ovary-specific protein. We found that the *igf3* mRNA level increased over time as ovarian follicular matured, indicating that Igf3 is a regulator of ovarian maturation in *E. coioides*. Igf3 promoted the expression of the sex differentiation-related genes such as *foxl2*, *dax1*, *cyp19a1a*, *cyp19a1b*, and *cyp11a1* and also promoted the expression of the reproduction-related gene, *lhr1*. This demonstrated that *igf3* plays a key role in sex differentiation and reproduction of *E. coioides*.

## Figures and Tables

**Table 1:** Primers used in this study.

Gene Symbol	GenBank Accession Number	Full Gene Name	Primer Sequence(5′-3′)	Amplification Efficiency (%)
*β-actin*	AY510710	Beta-actin	Forward: TTCACCACCACAGCCGAGA Reverse: TGGTCTCGTGGATTCCGCAG	100
*igf3*	KU743244.1	Insulin-like growth factor subtype 3	Forward: GTGTGTATGTTCTACTCCAC Reverse: TCCTTCTGTAAGCTTCCCTC	100
*foxl2*	KP688066.1	Forkhead box L2	Forward: CAGGTAGCCATAGCCGTCTTCT Reverse: CCTCCACCGACGCACTTC	99
*dax1*	HQ455522.1	Dosage-sensitive sex reversal adrenal hypoplasia critical region on chromosome x gene 1	Forward: CTGCCTCCACTACATCCAGT Reverse: TGCTGACGGTCCCTATAACG	98
*cyp19a1a*	EU239953.1	Cytochrome P450 family 19 subfamily member 1 a	Forward: GGAGACATTGTGAGAGTCTGGATC Reverse: TGACAGGTACATCCAGGAAGAGTC	99
*cyp19a1b*	JF420889.1	Cytochrome P450 family 19 subfamily member 1 b	Forward: TGACACCTGGCAAACAGTTC Reverse: GATGGTGTCGTCTTCCAGAG	97
*cyp11a1*	FJ807733.1	Cytochrome P450 family 11 subfamily a member 1 a	Forward: CTCCCTCCTGCTCTGTTGA Reverse: GCTGGCCTTGATGTCTTC	96
*lhr1*	HQ650770.1	Luteinizing hormone receptor 1	Forward: GACCTTCCCATTAATCCTGCATGG Reverse: CACGTTTCCAAAGCCTTCATCATCCTC	99
*fshr*	HQ650769.1	Follicle-stimulating hormone receptor	Forward: CGAGGCTGACCCTTACTTCC Reverse: GATCCAGATGAGGACCCGTA	98

## Data Availability

All relevant data are within the paper and its Supporting Information files.

## Supplementary Materials

The following supporting information can be downloaded at: https://www.mdpi.com/article/10.3390/ijms24032868/s1.
