# Supplementary figures and images for: The Expression Pattern of Insulin-Like Growth Factor Subtype 3 (igf3) in the Orange-Spotted Grouper Epinephelus coioides and Its Function on Ovary Maturation
Source: Int J Mol Sci. 2023 Feb 2;24(3):2868. doi: 10.3390/ijms24032868 (PMC9918221; doi:10.3390/ijms24032868)

Supplementary information

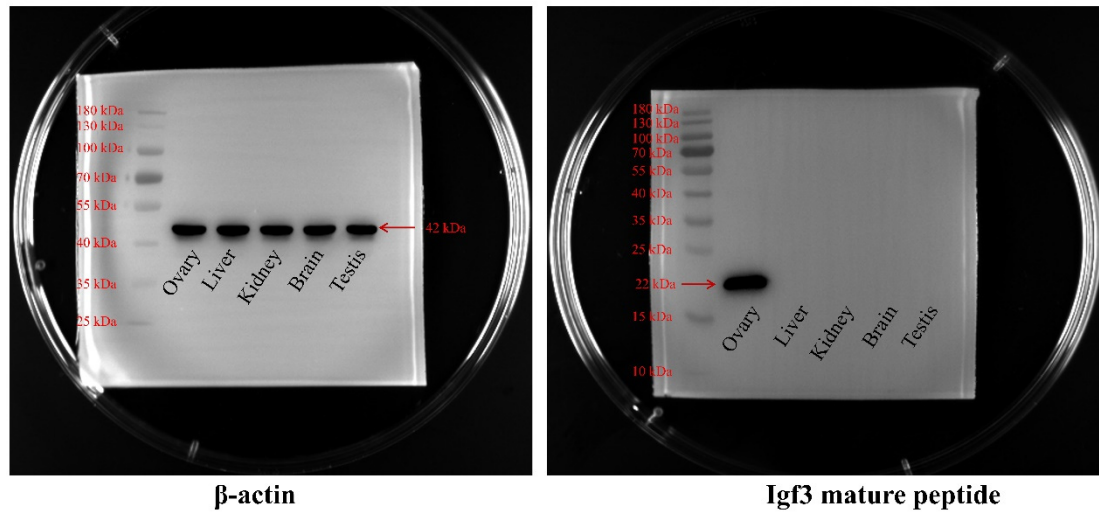

**Figure S1.** Original Western blot verification of Igf3.

Supplement: Supplementary file 1 [file ijms-24-02868-s001.zip › ijms-2060644-supplementary.pdf]
